# Supplementary figures and images for: The expression and clinical significance of GADD45A in breast cancer patients
Source: PeerJ. 2018 Aug 15;6:e5344. doi: 10.7717/peerj.5344 (PMC6098681; doi:10.7717/peerj.5344)

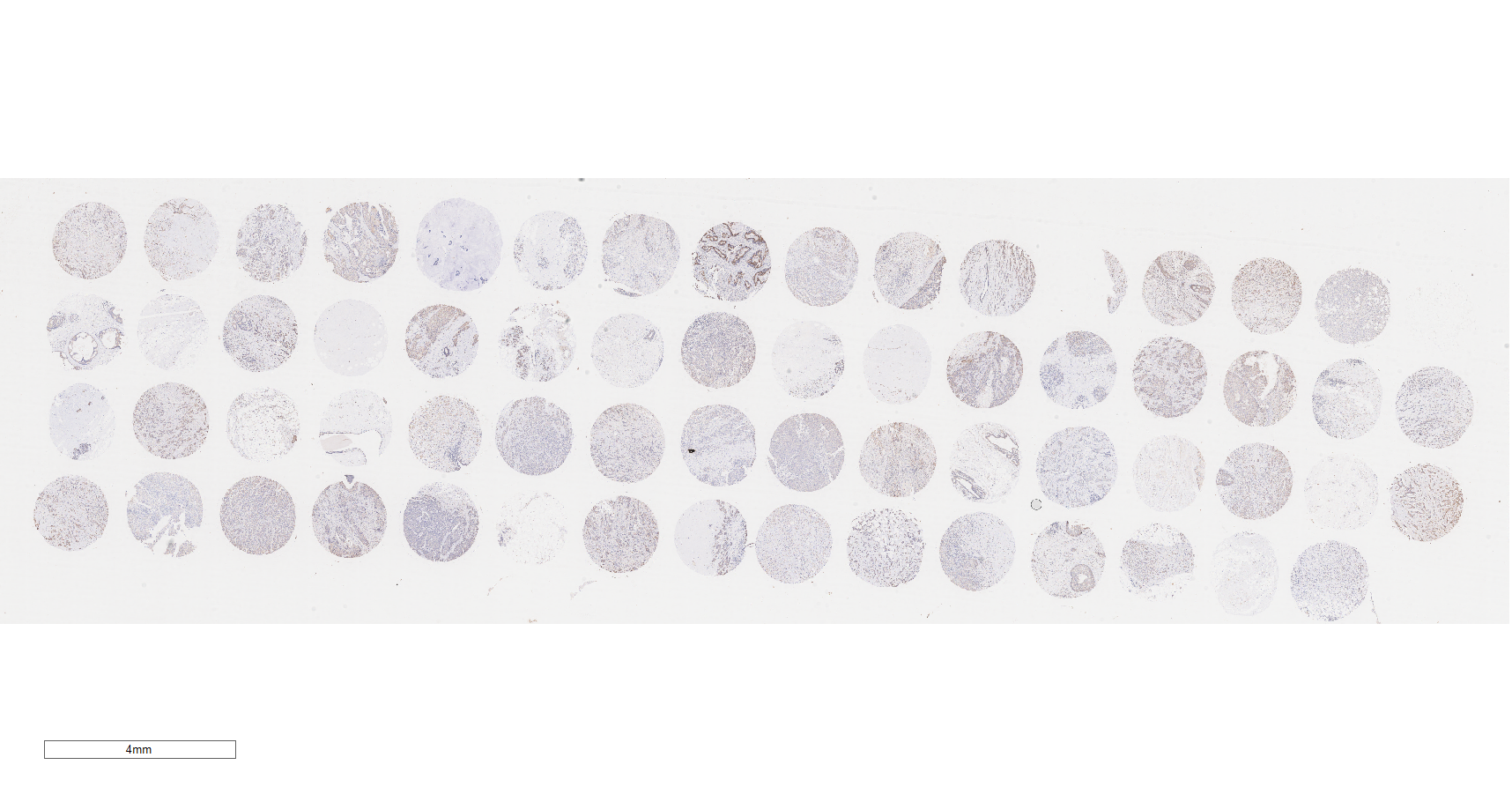

Supplement: Supplemental Information 1 [file peerj-06-5344-s001.tif]
